# Social capital's association with self-reported health outcomes in the rural south: variations by demographic subgroup

**DOI:** 10.3389/fpubh.2025.1706869

**Published:** 2026-02-06

**Authors:** Lauren M. Bigger, Robin E. McGee, Alexis Smith, Regine Haardörfer, April Hermstad, Michelle C. Kegler

**Affiliations:** Department of Behavioral, Social and Health Education Sciences, Emory Prevention Research Center, Rollins School of Public Health, Emory University, Atlanta, GA, United States

**Keywords:** health status, mental health, physical health, rural health, social capital

## Abstract

**Background:**

Limited information is known about how social capital is associated with self-reported measures of health in rural settings. Even less is known about how these associations may vary within demographic subgroups. While a large body of research demonstrates that social capital is associated with a variety of health outcomes, additional knowledge is needed to create appropriate interventions for rural communities in the United States.

**Methods:**

A mail-in population-level survey was conducted across six counties in the rural southern United States as part of a broader health equity initiative. Multivariable logistic regression models were run to assess how five domains of social capital (trust, diversity of interaction, reciprocity, civic engagement and voting behaviors) were associated with health status, physical distress and mental distress.

**Results:**

Social capital domains were positively associated with good health status, infrequent physical distress (defined as <14 days per month) and infrequent mental distress (defined as <14 days per month) for most subgroups. However, there were little to no significant findings among Black individuals and young adults.

**Conclusions:**

These findings suggest that social capital may not operate equally across demographic subgroups, particularly for Black individuals and young adults. Social capital may not be related for some subgroups and domains, or new measures may need to be developed to better represent how social capital operates. Public health practitioners should be particularly attuned to these groups as social capital interventions are trialed in rural communities.

## Introduction

There are growing disparities between rural and urban communities in a variety of health measures (1). A growing body of research provides evidence that better health outcomes and overall health status is by and large, associated with higher levels of social capital (2, 3) and that efforts to improve social capital can serve as a health promotion intervention, although the mechanics are still debated (4). Many of these studies, which demonstrate an association between social capital and health outcomes, have focused on urban populations and less is known about how social capital operates in rural settings and among various demographic groups (e.g., age, gender, racial identity, education). Rural communities are often studied as a monolith, flattening out the nuanced differences that exist among a single community. However, different subgroups (defined by age, gender, socioeconomic status and race) in rural populations may experience social capital in different ways. Therefore, our study specifically investigates the role of social capital on health across demographic subgroups in a rural, southern U.S. population to ascertain potential differential associations.

The field of social capital is diverse, and while there is no single agreed upon definition or measure for social capital, it is understood to be “features of social structures-such as levels of interpersonal trust and norms of reciprocity and mutual aid-which act as resources for individuals and facilitate collective action” [(5), p. 175]. The concept of social capital can be measured and operationalized at both the individual- and community-level and for this study we have chosen to operationalize it at the individual level.

A meta review of systematic reviews conducted by Ehsan and colleagues in 2019 summarized evidence suggesting that social capital may be a predictor of mental and physical health (6). However, these associations vary by the social capital domain, the setting or geographic location of the studies, and whether social capital is operationalized at the individual level, or a higher level such as neighborhood- or state-level. Another 2019 systematic review found that the direction of the associations between social capital and physical health also varied based on how physical health is operationalized. For example, studies with self-reported health as the outcome have had mixed results, while studies focused on cancer outcomes revealed a more clearly positive association (3).

The relationship between indicators of mental health and social capital are more robust and consistent than physical health. Studies have found a positive association between the social capital domain of trust and mental health outcomes, demonstrating that both generalized trust and trust in others can reduce the likelihood of depressive symptoms (7). A systematic review of social capital and mental illness concluded that the focus of studies on this topic has been on economically poor, urban populations, to the neglect of examining the association that social capital (including individual level social capital) has with mental illness in rural communities (8). A more recent systematic review conducted on neighborhood biopsychosocial health and neighborhood levels of social capital found that it did positively impact neighborhood health, but the study was global in nature and did not report on neighborhood characteristics (9).

While studies like these have examined a range of social capital domains, few have assessed variations in the associations between social capital domains and health across subgroups in a single study. This limits our understanding of the construct as a whole and what domains may be most salient and potentially health protective. Rather, the majority of research studies have focused on a single demographic group and health outcome, limiting our ability to understand the nuances within the broader population in a single setting. For example, a handful of studies have focused specifically on older adults and separately on young adults and youth and found that associations between the impact of social capital and health may vary by age. A 2017 systematic review of the impact of social capital interventions on the health of individuals 65 and older found a positive association between social capital and mental health (10). In another study of 65–75 year olds on the associations between social participation, interpersonal trust, and self-rated health, social participation, in particular, was found likely to be age-related; findings from this Finland-based study suggest that age may be more predictive of social capital than place (11). However, a scoping review focused on adolescents and young adults identified several studies that examined neighborhood social capital and depressive symptoms that mostly indicated an inverse association, and general health and mental health that mostly indicated a positive association (12). Studies on the positive impact of social capital on youth, young adults and adolescents commonly focus on outcomes tied to education, future educational attainment, or risky behaviors (e.g., sexual health and substance use) (13–15). Social capital for this group is frequently conceptualized at the community-level, analyzing the protective effects of a cohesive community, making it challenging to ascertain the role a young adult's perception of their own social capital has on their individual health outcomes (16).

Research focused on sex/gender has found that males and females in Italy may have different types and levels of social capital over the life course, with men having a peak of social capital in their mid-life whereas women's consistently declined (17). However, when evaluating the association of social capital with health-related outcomes, researchers found that women may reap more health benefits from increased social capital compared to men, particularly when measured at the neighborhood level (18, 19).

While the race or ethnicity of an individual can be used as a proxy measure or indicator of racialized discrimination or differential cultural-based engagement in community activities, very little research has been done on how the association between social capital and health outcomes vary by race (20). One study investigating whether the relationships between social capital and health status differs between Black and White individuals found that structural inequalities disproportionately negatively impact Black individuals and may inhibit the benefits conferred through social capital. Even when Black individuals had similarly high levels of social capital, the health benefits were weaker than those experienced by White individuals (21).

Finally, socioeconomic status, as measured by income and level of education, may also impact individual-level social capital and associated health status. Among the first to study social capital and health were Kawachi et al. (22) in their ecological study which explored associations between state levels of income inequality, social capital, and mortality. They found that social capital domains of trust and group membership were inversely associated with mortality rates. Additionally, states with greater income inequality also had lower social capital levels and higher rates of mortality (22). Additional research over the last two decades has found similar results. Another analysis, using data from a European sample, found that those with higher levels of education reported higher levels of well-being irrespective of their level of social capital. However, among those with lower education, increased levels of well-being were associated with higher levels of social capital (23).

The current body of research provides a base of knowledge that a higher level of social capital is associated with good health; however, the field is challenged to achieve consensus on the nature of these relationships due to the variation in measures used, and the settings and populations studied. In order to inform public health practice in rural populations, more detailed analyses are needed to understand the differential impact of individual-level social capital on health outcomes across demographics such as age, gender, race, and education in a population. To date, few studies have explored these multiple nuanced associations in a single setting. Our previous work found variation in the strength of associations by race between some measures of social capital and health, prompting us to further assess a wider variety of demographic characteristics and social capital domains (20). The present study hypothesizes that there are associations between the social capital domains of trust, diversity of interaction, reciprocity, civic engagement and voting behavior and overall health status, mental health and physical health in rural populations and that these associations differ by demographic characteristics.

## Methods and materials

The current study is a secondary analysis of data collected through a cross-sectional survey conducted as part of a coalition-led, rural health equity initiative in Georgia (24). Additionally, this analysis extends an earlier social capital analysis from the sample by further exploring demographic subgroups (20).

### Sampling and recruitment

This study used data from a cross-sectional population survey conducted early in the initiative that explored both behaviors and environments related to commonly prioritized areas (e.g., health care access and use, food access and healthy eating). The Emory University Institutional Review Board (IRB) determined that this study was a non-research program evaluation and therefore did not require IRB approval.

### Pertinent sampling details from the original health equity initiative

The sampling frame consisted of residential mailing addresses randomly selected from commercial lists which were purchased for each coalition county. Households in each coalition county received surveys that were tailored to the county's key priority areas, with input from coalition staff and a local evaluator. Data for this study are limited to the coalition counties (*N* = 6 out of the 11 coalitions in the full evaluation study) that chose to include the social capital module.

The baseline surveys were mailed to 5,558 households in waves between December 2018 and June 2019 across the six counties whose data are included in the present study. The mailing included a description of the study and incentive, in addition to a copy of the survey and a pre-stamped return envelope. The study was conducted in accordance with the Declaration of Helsinki and survey participants were provided with details of the study prior to their participation, including assurances of confidentiality and the voluntary nature of their participation. Survey respondents indicated informed consent by returning completed questionnaires. Over the course of a month, each household sequentially received the survey mailing, a postcard reminder and then a second survey mailing, depending on their response timing. Through these efforts, the response rate was 24.9%. A complete survey was one with at least 50% of the questions answered. Respondents received a $15 gift card for the completed survey. The survey was completed by 1,338 eligible respondents.

## Measures

### Variables and measures

*Social capital domains:* For this study, we assessed five domains of individual-level social capital: trust, diversity of interaction, civic engagement, reciprocity, and voting behavior. These domains are theoretically grounded in individual-level social capital definitions (25, 26).

*Trust*: Eight items were adapted from the SCBS (27) to measure the extent to which residents trust those within their social networks. Respondents were directed to consider how much they trust different groups of people: people in general, friends and family, people that live near you, people from a different racial/ethnic group than your own, people with different political views than your own, people from a different religion than your own, people with more or less education than you, and people with a lot more or a lot less money than you. Response options ranged from 3 to 0 and were “trust them a lot”, “trust them some”, “trust only a little”, “trust them not at all”. These items were summed to create a trust score (ranging from 0 to 24) where higher scores indicate having more trusting relationships.

*Diversity of interaction:* To capture the diversity of individual social networks, we adapted eight items informed by the SCBS. For this section, items asked how often the participant talks to or spends time with different groups of people, noting that these interactions could be in person, over the telephone, or through the internet or social media. Questions asked about their frequency of interaction with the same groups as above. Response options (3–0) were “very often”, “often”, “not very often”, and “not at all.” These items were summed to create a diversity of interactions score that could range from 0 to 24, where higher scores indicated that respondents have more frequent interactions with a diverse social network.

*Reciprocity*: Three items assessed respondents' notions of reciprocity in their respective community. We asked how much they agree or disagree (with response options on a Likert scale from 1 to 5, ranging from “strongly disagree” to “strongly agree”) with the following statements: “People around here are willing to help their neighbors,” “If I had to borrow $30 in an emergency, I could borrow it from a neighbor,” “If I were sick, I could count on my neighbors to shop for groceries for me.” These questions were summed to create a reciprocity score between 3 and 15 (28).

*Civic engagement:* To measure civic engagement, we included 10 items on respondents' participation in various civic engagement activities over the past 12 months (e.g. attending a political meeting or event, belonging to any groups, organizations, or associations, or attending religious services at least once per month). These items were also adapted from the SCBS and the U.S. Census Community Population Survey Civic Engagement Supplement (29). Response options were yes = 1 or no = 0. These ten items were summed to create a civic engagement score that could range from 0 to 10, with 10 indicating higher levels of engagement.

*Voting behavior:* Respondents were asked three questions from the SCBS about their voting behavior in the most recent presidential, midterm, and local elections. Response options were “yes,” “no,” and “don't know.” Responses were dichotomized to “yes” or "no,” recategorizing the “don't know” responses as “no,” as the intent for this question was to capture voting engagement. For analytical purposes we reduced these 3 behaviors to 1 variable by categorizing respondents as “yes” if they indicated having voted in all 3 elections.

## Health measures

*Health status:* We assessed self-rated general health status by asking “In general, would you say that in general your health is…” Response options were “excellent”, “very good”, “fair”, and “poor.” These were dichotomized such that excellent, very good and good responses were collapsed into one category labeled “good health,” and fair and poor were combined and labeled “poor health.” Self-rated health measures have been shown to be reliably predictive of objective physical and mental health as well as predictive of future negative health outcomes (30–32).

*Infrequent physical distress:* This measure was from the Center for Disease Control and Prevention's Health-Related Quality of Life (HRQOL)/Healthy Days measure (33) which asks respondents “Now thinking about your physical health, which includes physical illness and injury, for how many days during the past 30 days was your physical health not good?” We dichotomized the measure, where physical distress was defined as 14 or more days in which physical health was not good and <14 days as infrequent physical distress. In line with the “good health” measure, models were run to assess the positive which in this case was <14 days of “not good” physical health (infrequent physical distress).

*Infrequent mental distress:* This measure used the same source as physical distress, and asked respondents “Now thinking about your mental health, which includes stress, depression, and problems with emotions, for how many days during the past 30 days was your mental health not good?” We also dichotomized this measure, defining mental distress as 14 or more days in which mental health was not good or in which the respondent experienced “frequent mental distress” vs. <14 days of “not good” mental health, indicating infrequent mental distress. Again, models were run to assess reporting <14 days of “not good” mental health (infrequent mental distress).

*Demographics:* We included measures on both individual and household-level characteristics including age, gender, race and ethnicity, educational attainment, household income, and rurality adapting these common questions from the Behavioral Risk Factor Surveillance System (34) and the U.S. Census Bureau's American Communities Survey (35).

## Analysis

Data for this study were analyzed using SAS Version 9.4. Descriptive statistics were employed to examine the demographic characteristics of our study sample. We assessed the distributions of all of the social capital domains (independent variables) and health measures (outcomes) to through univariate analysis and histograms to characterize the sample. The mental and physical health measures were dichotomized in line with existing literature, and we chose to report on the health measures as a positive outcome (i.e. good health status, infrequent physical distress and infrequent mental distress) to aid in the ease of reporting, comparability with other studies and practical use of the findings.

The data were stratified by demographic characteristic and then a series of binary logistic regression models were used to estimate the association between each of the five measures of social capital and self-reported health status, infrequent physical and mental distress for each demographic group of interest: Gender (male/female), race (Black/White), education (college/no college), and age (18–34, 35–64, 65+). Adjusted models were run to include county, gender, race, level of education and age (if that was not the selected demographic characteristic for stratification) so that we could isolate the demographic of interest. We report on confidence interval and *p*-value to assess model fit. These adjusted multivariable models were run to quantify the associations between the five social capital domains and the following outcomes: odds of reporting “good health” compared to reporting fair/poor health status, the odds of reporting infrequent physical distress or frequent physical distress and the odds of reporting infrequent mental distress or frequent mental distress.

In several cases, mainly with the age variable, there was quasi-complete separation of data points detected in the original regressions due to low cell count. This was not unexpected, as we used data from a rural area where the younger population is under-represented. To accommodate the low cell count, while still assessing the various demographic segments of the population, we took the following approach. For the male group, there were few young men who reported negative health outcomes for good health status and physical distress and so we removed the youngest age group from that analytic data set. Similar problems arose in the young adult age set in general (age 18–34) and unadjusted logistic regression models were run for this subset for the health outcomes of good health status and infrequent physical distress. Additionally, in the subset of Black respondents, one county had very small cell sizes and after assessing that there were no qualitative differences between the counties across demographics in terms of outcome, that single small county was removed from that analytic data set for the outcome of infrequent mental distress.

## Results

Table 1 summarizes demographic characteristics of the full sample. All participants were 18 years old or older, with the majority being between 35–64 years of age. The majority of participants were women, White individuals, had at least some college education and participants were fairly evenly distributed across the counties of study.

**Table 1:** Population characteristics.

**Individual-level factors** ***N* = 1,338**	***N* (%)/Mean (SD)**	**% Good health**	**% Infrequent physical distress**	**% Infrequent mental distress**
Total population		75.85%	81.16%	86.29%
**Age**				
18–34	103 (7.7)	92.2%	91.2%	82.4%
35–64	677 (50.6)	74.6%	79.8%	83.4%
≥65	558 (41.7)	74.3%	80.9%	90.8%
**Gender**				
Male	441 (32.8)	75.7%	81.5%	90.9%
Female	902 (67.2)	76.7%	81.3%	84.2%
**Race**				
White, not of Hispanic origin	998 (77.3)	58.7%	81.8%	86.9%
African American/Black, not of Hispanic origin	293 (22.7)	63.4%	78.0%	84.7%
**Education**				
Some high school or less	146 (10.9)	56.3%	73.2%	83.5%
High school or GED	374 (27.8)	71.9%	78.8%	87.4%
Some college or technical school	418 (31.1)	77.7%	79.5%	84.7%
College graduate and above	407 (30.3)	85.8%	87.8%	88.8%
**County**				
County A	201 (14.6)	72.5%	80.7%	89.0%
County B	247 (18.0)	76.3%	79.8%	86.8%
County C	184 (13.4)	73.9%	81.5%	85.6%
County D	230 (16.7)	75.3%	79.2%	87.7%
County E	303 (22.1)	80.5%	83.0%	85.0%
County F	209 (15.2)	74.0%	82.2%	84.0%
**Social capital constructs**				
Trust	14.1 (5.1)			
Diversity of interaction	13.4 (5.0)			
Reciprocity	3.4 (1.1)			
Civic engagement	3.2 (2.8)			
Voting	2.3 (1.1)			
**Health measures**				
**Health status**				
Poor	67 (5.0)			
Fair	258 (19.1)			
Good	528 (39.2)			
Very good	394 (29.2)			
Excellent	101 (7.5)			
Physical distress	6.1 (9.4)			
Mental distress	4.4 (8.5)			

The results of the regression analyses are presented by dependent variable and then further organized by demographic characteristic (gender, race, education and age). The subsequent tables are organized by health outcome.

### Health status

*Gender:* As shown in table 2, there were significant associations between nearly all domains of social capital and good health status among both men and women. The highest estimate among men (age 35 and older as the lower age group was removed from this analytic data set due to low cell count) was voting whereby for a one unit increase in voting, the odds of reporting good health increased by a factor of 1.91 (95% CI: 1.13, 3.22). Among women, voting was not a significant predictor, and the highest domain was reciprocity where odds of reporting good health increased by a factor of 1.51 for a one-point increase in reciprocity (95% CI: 1.29, 1.76).

**Table 2:** Multivariable logistic regression associations between dimensions of social capital and good health status by demographic subgroup.

**Subgroup**	**Trust OR (CI)**	**Diversity of Interaction** **OR (CI)**	**Reciprocity OR (CI)**	**Civic Engagement** **OR (CI)**	**Voting OR (CI)**
**Gender**					
Male^†^	1.05^*^ (1.00, 1.11)	1.10^**^(1.03, 1.17)	1.28^*^(1.01, 1.62)	1.25^****^(1.13, 1.39)	1.91^*^ (1.13, 3.22)
Female	1.04^*^ (1.01, 1.08)	1.09^****^ (1.06, 1.13)	1.51^****^ (1.29, 1.76)	1.20^****^ (1.11, 1.29)	1.97 (1.39, 2.80)
**Race**					
White	1.06^****^ (1.03, 1.10)	1.11^****^ (1.07, 1.15)	1.66^****^ (1.42, 1.94)	1.28^****^ (1.18, 1.38)	2.28^****^ (1.61, 3.21)
Black	1.03 (0.95, 1.06)	1.02 (0.96, 1.08)	1.05 (0.84, 1.33)	1.09 (.98, 1.21)	1.40 (.82, 2.41)
**Education**					
College	1.08^*^ (1.01, 1.16)	1.08^*^ (.1.00, 1.17)	1.73^***^ (1.27, 2.34)	1.22 ^***^ (1.09, 1.37)	1.80 (.88, 3.66)
No College	1.04^**^ (1.01, 1.08)	1.09^****^ (1.05, 1.12)	1.37^****^ (1.19, 1.58)	1.23^****^ (1.15, 1.32)	2.13^****^ (1.56, 2.91)
**Age**					
18–34 years^~^	1.09 (0.94, 1.25)	0.98 (0.56, 1.12)	1.81 (0.94, 3.49)	0.94 (0.74, 1.19)	0.91 (0.22, 3.89)
35–64 years	1.05^*^ (1.01, 1.10)	1.08^****^ (1.04, 1.13)	1.57^****^ (1.30, 1.98)	1.22^****^ (1.12, 1.32)	2.31^****^ (1.54, 3.48)
65+ years	1.03 (0.99, 1.08)	1.10^****^ (1.05, 1.15)	1.29^**^ (1.06, 1.56)	1.25^****^ (1.23, 1.37)	1.77^**^ (1.15, 2.72)

Logistic regressions control for county, age, race, employment, gender, and education (if it is not the selected characteristic for stratification).

^*^Indicates *p* value <0.05 | ^**^ <0.01 | ^***^ <0.001 | ^****^ <0.0001.

^†^Gender: removed the lower age group (18–34) from the male model here due to quasi-separation of the data.

~Age: 18–34 was run as an unadjusted model due to the small cell size for the poor health outcome.

*Race:* All five social capital domains were positively and significantly associated with the odds of reporting good health among White individuals, but not among Black individuals. The highest estimates among White residents were for voting (2.28, 95% CI: 1.61, 3.21) and reciprocity (1.66, 95% CI: 1.42, 1.94).

*Education:* Four out of the five social capital domains were positively and significantly associated with good health status (voting was not) among those with a college education with reciprocity as the highest estimate. For a one unit increase in reciprocity, the odds of reporting good health increased by a factor of 1.73 (95% CI: 1.27, 2.34). Among those without a college degree, all the domains were positively and significantly associated. Reciprocity was also relatively high for this group where for a one unit increase in reciprocity, the odds of reporting good health increased by a factor of 1.37 (95% CI: 1.19, 1.58). In contrast to those with a college education, voting was significantly and positively associated with good health status for those without a college degree where for a one unit increase in voting, the odds of reporting good health increased by a factor of 2.13 (95% CI: 1.56, 2.91).

*Age:* Due to low cell counts, this model was unadjusted, and no domains of social capital were found to be associated with good health status for those who were between 18 and 34 years of age. The remaining age subgroups were run as adjusted models for greater specificity. In contrast, all five social capital domains were positively and significantly associated with health status among those who were 35–64 years of age, with the strongest associations found for voting (2.31, 95% CI: 1.54, 3.48) and reciprocity (1.57, 95% CI: 1.30, 1.98) respectively. Among those ages 65 and older, trust was not significantly associated with health status although the other four domains were positively associated. As in the middle-age group, reciprocity (1.29, 95% CI: 1.06, 1.56) and voting (1.77, 95% CI: 1.15, 2.72) again showed the largest associations.

### Infrequent physical distress

*Gender:* In Table 3, among men, only trust, diversity of interactions and reciprocity had significant results. For a one unit increase in reciprocity, the odds of reporting infrequent physical distress increased by a factor of 1.38 (95% CI: 1.06. 1.80). Due to no 18–34-year-old men reporting physical distress, they were removed from the analytic data set for this model. There were significant associations between four domains of social capital and infrequent physical distress among women. In this analysis, reciprocity also had the highest coefficient, for a one-unit increase, the odds of reporting infrequent physical distress increased by a factor of 1.52 (95% CI: 1.28, 1.81).

**Table 3:** Multivariable logistic regression associations between dimensions of social capital and *infrequent* physical distress per by demographic subgroup.

**Subgroup**	**Trust OR (CI)**	**Diversity of interaction OR (CI)**	**Reciprocity OR (CI)**	**Civic engagement OR (CI)**	**Voting OR (CI)**
**Gender**					
Male^†^	1.08^*^ (1.02, 1.14)	1.10^**^ (1.03, 1.17)	1.38^*^ (1.06. 1.80)	1.10 (0.99, 1.22)	1.32 (0.72, 2.41)
Female	1.03 (0.99, 1.07)	1.10^****^ (1.05, 1.14)	1.52^****^ (1.28, 1.81)	1.13^**^ (1.04, 1.22)	1.47^*^ (1.00, 2.15)
**Race**					
White	1.07^***^ (1.03, 1.10)	1.12^****^ (1.08, 1.16)	1.64^****^ (1.38, 1.93)	1.20^**^ (1.04, 1.21)	1.88^***^ (1.31, 2.70)
Black	0.98 (0.92, 1.05)	1.04 (0.97, 1.11)	1.15 (0.87, 1.51)	1.08 (0.96, 1.22)	0.67 (0.35, 1.27)
**Education**					
College	1.07 (1.00, 1.16)	1.08 (1.00, 1.16)	2.08^****^ (1.48, 1.64)	1.18^**^ (1.05, 1.34)	1.90 (0.91, 3.96)
No College	1.04^*^ (1.01, 1.08)	1.10^****^ (1.06, 1.15)	1.40^****^ (1.19, 1.63)	1.10^*^ (1.02, 1.18)	1.38 (0.97, 1.94)
**Age**					
18–34 years^~^	1.04 (0.91, 1.20)	1.03 (0.90, 1.16)	1.84 (0.98, 3.46)	0.87 (0.70, 1.09)	0.43 (0.10, 1.82)
35–64 years	1.05 (1.00, 1.09)	1.10^****^ (1.05, 1.15)	1.57^****^ (1.30, 1.91)	1.16^***^ (1.06, 1.26)	1.39 (0.91, 2.15)
65+ years	1.06^*^ (1.01, 1.12)	1.11^***^ (1.05, 1.17)	1.36^**^ (1.09, 1.70)	1.07 (0.97, 1.18)	1.57 (0.96, 2.58)

Logistic regressions control for county, age, race, employment, gender, and education (if it is not the selected characteristic for stratification).

^*^Indicates *p* value <0.05 |^**^, <0.01 | ^***^ <0.001 | ^****^ <0.0001.

^†^Gender: removed the lower age group (18–34) from the male model due to quasi-separation of the data.

~Age: 18–34 was run as an unadjusted model due to the small cell size for the poor health outcome.

*Race:* Among White individuals, there were significant associations between all domains of social capital and infrequent physical distress. Voting behavior had the strongest outcome where for a one unit increase in voting, the odds of reporting infrequent physical distress increased by a factor of 1.88 (95% CI: 1.31, 2.70). Among Black individuals there were no significant results between social capital domains and infrequent physical distress reported.

*Education:* The results varied for education level. Reciprocity and civic engagement were both significantly associated with infrequent physical distress for both those with and without a college education. However, among those without college education, trust and diversity of interaction were also associated with infrequent physical distress. The strongest association for both categories of education was reciprocity where the odds of reporting infrequent physical distress among those with college education increased by a factor of 2.08 (95% CI: 1.20, 1.64) but only by 1.40 (95% CI: 1.19, 1.63) for those without a college education.

*Age:* There were no significant results among those aged 18–34 years. Due to low cell counts, this model was run without adjusting for county, race, gender or education. The strongest association was found among those aged 35–64 years where for a one unit increase in reciprocity, the odds of reporting infrequent physical distress increased by a factor of 1.57 (95% CI: 1.30, 1.91), but diversity of interaction, and civic engagement were also positively and significantly associated. Trust, diversity of interaction and reciprocity were associated with infrequent physical distress among those 65 years of age and older with reciprocity having the strongest association where for a one unit increase in reciprocity the odds of reporting infrequent physical distress increased by a factor of 1.36 (95% CI: 1.09, 1.70).

### Infrequent mental distress

*Gender:* There were significant associations between all domains of social capital and infrequent mental distress among women, but only three domains were significant for men (Table 4). Among men, trust, diversity of interaction and reciprocity were found to be significantly associated with reporting infrequent mental distress in the past month. In contrast to the models assessing the physical health outcome, younger men *did* report mental distress, so this model represents the full age range of participants. Reciprocity was the highest estimate, where for a one unit increase in reciprocity, the odds of reporting infrequent mental distress increased by a factor of 1.48 (95% CI: 1.04, 2.10). Among women, voting had a high coefficient, for a one unit increase in voting, the odds of reporting infrequent mental distress increased by a factor of 2.60 (95% CI: 1.72, 3.94).

**Table 4:** Multivariable logistic regression associations between dimensions of social capital and *infrequent* mental distress per by demographic subgroup.

**Subgroup**	**Trust OR (CI)**	**Diversity of interaction OR (CI)**	**Reciprocity OR (CI)**	**Civic Engagement OR (CI)**	**Voting OR (CI)**
**Gender**					
Male	1.12^**^ (1.04, 1.20)	1.10^*^ (1.01, 1.19)	1.48^*^ (1.04, 2.10)	1.16 (1.00, 1.35)	1.68 (.77, 3.67)
Female	1.10^****^ (1.06, 1.15)	1.13^****^ (1.08, 1.18)	1.57^****^ (1.31, 1.89)	1.15^**^ (1.06, 1.26)	2.60^****^ (1.72, 3.94)
**Race**					
White	1.11^****^ (1.06, 1.15)	1.12^****^ (1.08, 1.17)	1.61^****^ (1.34, 1.94)	1.21^****^ (1.10, 1.31)	2.70^****^ (1.78, 4.10)
Black^†^	1.09^*^ (1.01, 1.17)	1.09^*^ (1.01, 1.18)	1.47^*^ (1.06, 2.04)	1.08 (.94, 1.24)	1.70 (.82, 3.50)
**Education**					
College	1.07 (.99, 1.15)	1.13^**^ (1.04, 1.22)	1.60^**^ (1.13, 2.18)	1.18^*^ (1.04, 1.34)	2.39^*^ (1.15, 4.99)
No College	1.12^****^ (1.07, 1.16)	1.12^****^ (1.07, 1.16)	1.59^****^ (1.31, 1.93)	1.16^**^ (1.06, 1.27)	2.34^****^ (1.56, 3.53)
**Age**					
18–34 years	1.19^*^ (1.02, 1.38)	1.11 (.98, 1.25)	2.5^*^ (1.17, 5.56)	1.06 (.85, 1.32)	1.76 (.53, 5.84)
35–64 years	1.11^****^ (1.06, 1.16)	1.12^****^ (1.06, 1.17)	1.68^****^ (1.36, 2.07)	1.21^***^ (1.10, 1.34)	2.89^****^ (1.79, 4.66)
65+ years	1.11^**^ (1.04, 1.18)	1.13^**^ (1.05, 1.22)	1.32 (.99, 1.77)	1.12 (.97, 1.28)	1.95^*^ (1.01, 3.74)

Logistic regressions control for county, age, race, employment, gender, and education (if it is not the selected characteristic for stratification).

^*^Indicates *p* value <0.05 | ^**^ <0.01 | ^***^ <0.001 | ^****^ <0.0001.

^†^Race: removed participants from county 9 due to quasi-separation of the data.

*Race:* All of the social capital domains were significantly and positively associated with infrequent mental distress among White respondents. The highest estimate among White individuals was for voting; for a one unit increase in voting, the odds of reporting infrequent mental distress increased by a factor of 2.70 (95% CI: 1.78, 4.10). There were three social capital domains significantly associated with increased odds of reporting infrequent mental distress among Black respondents, with reciprocity as the strongest predictor. For a one unit increase in reciprocity, the odds of reporting infrequent mental distress increased by a factor of 1.47 (95% CI: 1.06, 2.04). As a reminder, one county was removed from the analytic data set focused on Black respondents due to low cell counts with the outcome of interest.

*Education:* All five social capital domains were positively and significantly associated with infrequent mental distress among those without college education, with voting having the strongest association. For a one unit increase in voting, the odds of reporting infrequent mental distress increased by a factor of 2.34 (95% CI: 1.56, 3.53). For those with a college degree, there was no association seen for the domain of trust. However, the other domains were significantly associated and voting also had the highest estimate where the odds of reporting infrequent mental distress increased by a factor of 2.39 (95% CI: 1.15, 4.99).

*Age:* Unlike the other health outcomes, two social capital domains were found to be associated with infrequent mental distress for those between 18 and 34 years of age, trust (1.19, 95% CI: 1.02, 1.38) and reciprocity (2.50, 95% CI: 1.17, 5.56). In contrast, all five social capital domains were positively and significantly associated with infrequent mental distress among those who were 35–64 years of age with the strongest associations found for voting (2.89, 95% CI: 1.27, 4.66). Among those ages 65 and older, 3 domains were significantly associated, including trust, diversity of interaction and voting with voting having the highest estimate. For a one-point increase in voting, the odds of reporting infrequent mental distress increased by 1.95 (95% CI: 1.01, 3.74).

## Discussion

This study on social capital and health status, physical health and mental health among southern rural adults showed that, as hypothesized, all social capital domains were associated with health measures for some demographic groups. Across some, but not all demographics, health status was positively and significantly associated with all domains of social capital. Again, these associations were not commonly present for Black residents, nor those aged 18–34 years. In general, stronger associations were found for reciprocity, civic engagement and voting while there were more moderate associations for trust and diversity of interaction. On the whole, infrequent physical distress was positively and significantly associated with all domains of social capital. However, these associations were not present for Black residents, nor those aged 18–34 years. In general, the strongest associations were found for reciprocity. Infrequent mental distress was largely positively and significantly associated with all domains of social capital and in general the strongest associations were found for voting.

Strikingly, for Black individuals, only three associations between social capital and infrequent mental distress (trust, diversity of interaction, and reciprocity) were significant, suggesting that by and large, these measures of social capital may not capture meaningful domains of social capital among this population. To that end, Gilbert et al. (36) argue for the development of measure of Black social capital to delineate the ways in which structural racism requires Black individuals to leverage and use social capital differently than White individuals. Others have similarly suggested that structural racism has resulted in the measures of social capital prioritizing the experiences of White individuals. Researchers have posited that this has resulted in the measures neglecting to capture what social capital looks like among racialized and ethnic minorities and their communities (37). As with measures such as self-rated health, many of the oft used measures have been validated on majority White samples; thus, they are not always as relevant and valid for other ethnic and racial minority groups (38–40). The same can be suggested for the measures of social capital and the extent to which they are both valid and reliable for Black communities. It is possible that they do not fully reflect the strengths, resources, and strategies employed by Black residents to be engaged in their community and welfare of their neighbors.

Similarly, there were only two significant findings for those 18–34 years of age, which were for trust and reciprocity and infrequent mental distress. Other studies have similarly found associations between mental distress and social capital among young people (12). However, limited research has considered the relevance of current measures of social capital for young adults, who are often managing several transitions. Young adults may be finishing school, looking for jobs, and beginning to live independently, which may alter opportunities to maintain trusted social relationships (41). Young adults in rural areas may decide to leave their rural community for different jobs or educational opportunities disrupting the social networks formed when they were younger. Additionally, their relationships with neighbors and other community members may not be as established as compared with older adults, and they may focus more on their own family as they navigate early parenthood. With these transitions, young adults may have fewer opportunities to volunteer and participate in groups. Lower civic engagement is documented for voting patterns, which are typically lower among young adults, and even more so in rural areas (42). Across various components of social capital, the life experiences of young adults may compromise the strength of their individual social capital, which may partly explain the findings of fewer social capital dimensions being significantly related to health outcomes among younger individuals.

Our study benefits from being based in a very specific location, providing a focused picture on the role of social capital on health outcomes in rural Georgia. However, this may limit the generalizability of our findings to other settings, regions of the U.S., and other countries. Additionally, as a cross-sectional study, we cannot examine causal relationships between the studied constructs. Finally, in regards to the measures that we used, social capital was operationalized at the individual-level, therefore findings may not apply to similar social capital domains measured at the neighborhood-level nor higher community-levels and all measures in the survey were self-report, so both the social capital predictors and health outcomes are subject to bias.

This study also highlights the methodological complexities and challenges inherent in studying rural populations which align with challenges noted by others (43). As detailed above, it is important to study the demographic facets of rural communities, but this further breaks down the already small sample size into smaller analytical data sets, making traditional multivariable regression approaches challenging. The sample size may reduce the statistical power in our models and can impact the effect size. A limitation of this study is that the small sample sizes introduce bias to our findings and limit the overall generalizability. A detailed understanding of the population is necessary to make sound analytic choices, and our study team did benefit from serving as the external evaluators of the broader parent study making us familiar with the study population. While our study adds to the body of research indicating repeated associations between indicators of social capital and health outcomes, our work also points to the need for further specialized research on social capital across the less represented demographic groups in rural communities, which in this study were those aged 18–34, (particularly men in that age range) and Black individuals. Future studies could focus on these populations more centrally, with stratified sampling efforts, to better understand how these social capital domains reflect their health outcomes. Qualitative studies could be particularly beneficial to ascertain what survey questions may better capture whether and how social capital operates for certain population groups, especially in rural communities. As other studies have recommended (4) public health professionals may consider strengthening social capital as an approach to improve health outcomes. Our study recommends that particularly in rural communities, it is important account for differential ways in which trust, diverse social interactions, reciprocity, civic engagement and voting behaviors may play out across myriad demographic subgroups, particularly among Black individuals and young adults. Qualitative studies or focus groups could be particularly beneficial to ascertain how social capital operates for these population groups in general and especially in rural communities.

## Data Availability

The datasets used for the current study are available from the corresponding author upon reasonable request.
